# Whole-Genome Sequence of French Clinical Olivibacter jilunii Strain P8502

**DOI:** 10.1128/MRA.00701-19

**Published:** 2019-08-01

**Authors:** Hussein Anani, May Khodor, Didier Raoult, Pierre-Edouard Fournier

**Affiliations:** a Aix Marseille Université, Institut de Recherche pour le Développement (IRD), Service de Santé des Armées, AP-HM, UMR Vecteurs Infections Tropicales et Méditerranéennes (VITROME), Institut Hospitalo-Universitaire Méditerranée Infection, Marseille, France; b Institut Méditerranée Infection, Marseille, France; c Aix-Marseille Université, Institut de Recherche pour le Développement (IRD), UMR Microbes Evolution Phylogeny and Infections (MEPHI), Institut Hospitalo-Universitaire Méditerranée-Infection, Marseille, France; d Special Infectious Agents Unit, King Fahd Medical Research Center, King Abdulaziz University, Jeddah, Saudi Arabia; Georgia Institute of Technology

## Abstract

In 2013, Olivibacter jilunii was reported as a bacterial species isolated from contaminated soil. In 2018, a clinical strain from the same species was isolated from the rectal swab of a Hajj pilgrim. Genome sequencing yielded 6,704,032 bp, with 41.2% G+C content, 5,406 protein-coding genes, and 54 predicted RNA genes in strain P8502.

## ANNOUNCEMENT

In 2007, Ntougias et al. proposed the creation of a novel bacterial genus, Olivibacter gen. nov. (1), within the family *Sphingobacteriaceae* in the phylum *Bacteroidetes* (2). Currently, this genus includes nine species with validly published names (3), as follows: Olivibacter sitiensis, the first species of the *Olivibacter* genus that was isolated from alkaline olive oil mill wastes in the region of Sitia, Crete in Greece (1), O. soli, O. ginsengisoli, O. terrae (4), O. oleidegradans (5), O. jilunii (6), O. composti (7), O. domesticus (8), and O. ginsenosidimutans (8). In 2018, we isolated from a fecal sample of a healthy Hajj pilgrim strain P8502 using the culturomics approach, an approach based on the use of a large panel of growing conditions (9–11). Growth was obtained after 24 h of culture in 5% sheep blood-enriched Columbia agar (bioMérieux, Marcy l’Etoile, France) in a strict aerobic atmosphere at 37°C. This bacterium exhibited 99.53% 16S rRNA sequence similarity with *O. jilunii* strain 14-2A^T^ (=KCTC23098^T^ =CCTCAB2010105^T^), its closest phylogenetic neighbor. *O. jilunii* strain 14-2A^T^ was isolated during the study of culturable heterotrophic bacteria in dichlorodiphenyltrichloroethane (DDT)-contaminated soil collected from a DDT production plant in Jiangsu, China (6). In our laboratory, DNA of *O. jilunii* strain P8502 was extracted using an EZ1 biorobot with the EZ1 DNA tissue kit (Qiagen). DNA was quantified by a Qubit assay with the high**-**sensitivity kit (Life Technologies, Carlsbad, CA, USA) at 0.2 μg/μl and was sequenced with the MiSeq platform (Illumina, Inc., San Diego, CA, USA). The DNA was fragmented and amplified by limited PCR (12 cycles), introducing dual-index barcodes and sequencing adapters. After purification on AMPure XP beads (Beckman Coulter, Inc., Fullerton, CA, USA), the libraries were normalized and pooled for sequencing on the MiSeq platform. Paired-end sequencing and automated cluster generation with dual**-**indexed 2 × 251-bp reads were performed during a 40-h run. Total information of 8.2 Gb was obtained from a 1,207,000/mm^2^ cluster density with a cluster passing quality control filters of 89.3% (10,507.2 passed filtered reads). Reads were quality checked using FastQC and trimmed using Trimmomatic version 0.36.6 (12). MiSeq reads were assembled using the SPAdes version 3.5.0 software (13). The option “careful” was used in order to reduce the number of mismatches and short indels. Default parameters were applied for k values, i.e., k-mer values of 127, 99, 77, 55, 33, and 21. SSPACE (14) and GapFiller (15) were used to combine contigs, using default parameters. The draft genome sequence of *O. jilunii* strain P8502 is composed of 23 contigs (*N*_50_, 537,034 bp; *L*_50_, 4) and 21 scaffolds (*N*_50_, 643,697 bp; *L*_50_, 3), for a total of 6,704,032 bp with 42.1% G+C content. Annotation using Prokka (version 1.12) (16) predicted 5,524 genes, including 5,406 protein-coding genes, 3,498 of which (63.32%) are assigned to clusters of orthologous groups. In addition, 51 RNA genes were detected (6 rRNAs and 45 tRNAs). In addition, 43 resistance genes are predicted, including 3 genes associated with antibiotic resistance (tetracycline). No toxin/antitoxin module or bacteriocin-associated gene could be found.

### Data availability.

The draft genome and read sequences (raw sequences) of *O. jilunii* strain P8502 have been deposited at GenBank/EBI under the accession numbers UWUA01000001 to UWUA01000023 and ERR3393031, respectively.
